# The level of safety standards in implementing therapeutic and caring procedures by emergency department personnel

**DOI:** 10.1002/nop2.196

**Published:** 2018-09-14

**Authors:** Motahareh Musavi Ghahfarokhi, Leila Masoudiyekta, Nasrin Khajeali

**Affiliations:** ^1^ School of Nursing and Midwifery Dezful University of Medical Sciences Dezful Iran

**Keywords:** emergency department, personnel performance, safety standards, therapeutic and caring procedures

## Abstract

**Aim:**

The present study aimed to determine the level of applying safety standards in treatment and therapy processes.

**Design:**

In the present descriptive study, 120 cases of nasogastric intubation, bladder catheterization and intramuscular and intravenous injections done by emergency staff were randomly selected.

**Methods:**

The data were collected by a two‐section checklist including demographic features and four sheets of observation. Then, the data were analysed based on descriptive statistics.

**Result:**

The results indicated that the level of compliance with safety standards was 63.3% in intramuscular injection, 86.7% for intravenous injection, 90% for bladder catheterization and 80% for nasogastric intubation. In addition, no statistically significant difference was observed between these processes and the variables such as ages, education and experience and work shift.

## 1 INTRODUCTION

The Health, Safety and Wellbeing in Healthcare Partnership Group (HSWPG) (formally known as the Partnership of Occupational Safety and Health in Healthcare (POSHH) has developed a set of standards with the support of the Health and Safety Executive. They pulled together the legal requirements and guidance to help organizations to comply with “goal setting” legislation. In addition, they provided practical pointers and signposting for meeting appropriate standards in key areas in workforce health and safety (Tracey & Sunley, 2013). Medical errors are regarded as the serious problems in public health and a threat for safety of patients since safety plays a pivotal role in health and treatment (Grober & Bohnen, 2005). Further, the nurses’ errors could be harmful for their profession, along with damaging the patients (Tang, Sheu, Yu, Wei, & Chen, 2007). Nurses are considered as the main group of healthcare providers in the hospital, who are generally closer to patients than other clinicians and spend most of their time in the patient care departments. As they oversee, coordinate and provide care, nurses are well positioned to strengthen the safety net for patient care in hazardous hospital environments. Given the integral role which nurses play in promoting their patients’ safety, more evaluation of the link between nursing work and patient safety is warranted (Vaismoradi, Salsali, & Marck, 2011).

The Human Right Prism of Europe in 2004 declared that each person has the right to be benefited from treatment and healthcare services according to the predetermined standards and he should be kept away from the damages due to impaired service and doctors’ and nurses’ errors (Eleftheriola, 2007). Regarding the implication of treatment‐caring services, injection is one of the main actions in the emergency department, leading to irrecoverable consequences if the safety principles are not complied, among which hospital infections and blood transmitted diseases are highlighted (Potter & Perry, 2003).

Based on a review of 30 national or multicentre point prevalence surveys in 19 countries conducted between 1996 and 2007, which included a total of 837,450 patients, European Centre for Disease Prevention and Control (ECDC) estimated the prevalence of hospital acute infections in emergency unit acute care hospitals to be on average of 7.1% during 2008 (Zarb et al., 2012). In addition, the exposure to sharp wastes is a potential risk for medical staff, especially nurses in hospitals. Needle‐stick and other percutaneous injuries cause the greatest risk of occupational transmission for serious bloodborne infections such as hepatitis B virus (HBV), hepatitis C virus (HCV) and human immunodeficiency virus (HIV) to healthcare workers (HCW) and patients (Centers for Disease Control & Prevention [Link] Lori, McCullagh, Krueger, & Oteng, 2016). According to the World Health Organization (WHO), 40% HCV and HBV and 2.5% HIV spread among healthcare workers through their exposure to needle‐stick injury (Mulanovich, Lescano, Gonzaga, & Blazes, 2007). In Iran, according to the studies related to the prevalence of these diseases, it is <1.7% for HBV (Poorolajal & Majdzadeh, 2009), <1% for HCV (Alavian, Adibi, & Zali, 2005) and 2 per 100,000 people for HIV (Miraghajani, Esmaillzadeh, Najafabadi, Mirlohi, & Azadbakht, 2012). According to Zeighami et al. (2014), the nurses in the emergency department are exposed to needle stick almost three times more than men due to the high volume of work. Fischer et al. (2010) confirmed that unsafe injection and reusing syringe and nonsterile needles are responsible for contacting among 8–16 million people with hepatitis B virus and 2.3–4.7 million people with hepatitis C virus. Based on the results of a study about patient safety and quality of care from the developing countries, four inter‐related safety and quality concerns including the risk of patient infection in healthcare delivery, medications errors/use, the quality and provision of maternal and perinatal care and the quality of healthcare provision were emphasized (Syed Abdul, Iqbal, & Li, 2015). HIV cases in Iran are estimated at 7,850 people and more than 60% of HIV cases in Iran are related to injecting drug‐addicted people according to the last statistics (Askarian, Aramesh, & Palenik, 2006). The contact of skin and mucus with contaminated needle and misuse or not applying protection means such as gloves are considered as the most common reason for HIV transmission in hospital (Poorolajal & Majdzadeh, 2009). In another study, Hazrati, Vahedi, and Salami (2013) indicated that intravenous injections in Tehran were performed well among 16% of the cases, which was evaluated as acceptable among 66% of cases while it was assessed as unacceptable among 18% of the cases. Thus, it is necessary to prevent blood‐transmitted disease. Safe injection and application of correct methods are among the main prevention methods for transmitting these disease and the other side effects of unsafe injection (Dentinger, Pasat, Popa, Hutin, & Mast, 2004).

Urinary tract infections are considered as one of the most common health care‐related infections in United States (Nicolle, 2014). These infections compose more than 40% of hospital infections (Chant, Smith, Marshall, & Friedrich, 2011). Unnecessary placement and continued use of urinary catheters remain common in hospitalized patients, especially among vulnerable populations such as older adults (Fakih et al., 2010). The risk exists when the patient reaches the hospital in the emergency department (ED) on both the intensive care unit (ICU) and the medical surgical floor, or in the operating room (OR) (Meddings & Saint, 2011). In addition, the abundance of fungi and bacteria in the patients’ urine with fully catheters in Shariati Hospital of Tehran was 25% and 38% among men and women, respectively (Pakshir, Moghadami, Emami, & Kordbacheh, 2004). In another study conducted in three selected hospitals of Iran by Farzianpour et al. (2014), the most invasive procedures performed on the patients afflicted with hospital infections included surgical operations, urinary catheters, venous catheters, tracheal tube, suction, ventilator and venous feeding, respectively. Thus, hospital infections can be substantially reduced through education (Farzianpour et al., 2014). According to Hampton (2004), improper locating on catheters is regarded as the most important risk factor. The recommendations given by the National Institute for Health and Care Excellence (NICE) for avoiding health care‐associated infections are based on hospital environmental hygiene, hand hygiene, the use of personal protective equipment (PPE), the safe use and disposal of sharps and asepsis principles (Loveday et al., 2014).

Further, nasogastric intubation is considered as another common process in hospital. Caring for the client with feeding tube generally involves maintaining tube patency, clearing any obstruction, providing adequate hydration, dealing with common formula related problem and preparing the client for home care (Timby & Smith, 2005). Patients with critical diseases and those needing artificial aspiration are prone to complications due to improper intubation in airways (Sorokin & Gottlieb, 2006). More than 50% of improper nasogastric intubation occurs in the patients with intratrachea tubes or tracheostomy (Sorokin & Gottlieb, 2006). Marderstein, Simmons, and Ochoa (2004) reported that 38% had serious pulmonary complications among 57 patients with mechanical ventilation who had impaired nasogastric intubation. In another study in Iran, Asefzade et al. (2013) indicated that the status of patient safety in education and treatment centres of Rasht city was unsatisfactory. However, Janghorbani, Raisi, Dehghani, and Mousavi (2011) in their study on the status of Shahid Beheshti Hospital operating room safety indicated that the level of patient safety standards was desirable. Finally, service quality enhancement in emergency department needs proper knowledge on the current condition and the evaluation of the problems in this sector (Jalalinia, Zakeri Moghadam, & Kazemnejad, 2006).

Regarding high sensitivity of emergency department as a high‐risk sector, any negligence in compliance with standards and principles can lead to irrecoverable consequences in the health and safety of patients and staff, compensation in the hospitals and the reduction in hospital official evaluation score.

## 2 AIM OF THE STUDY

Regarding the importance of patients’ and staff's safety, the present was performed to determine the level of applying safety standards in nasogastric intubation, bladder catheterization and intramuscular and intravenous injections done in emergency department in 2015.

## 3 METHOD

### 3.1 Participants

This is a descriptive and cross‐sectional study conducted for determining the level of compliance with safety principles in a treatment process including nasogastric intubation, bladder catheterization and intramuscular and intravenous injections in emergency department of educational hospital related to Dezful University of Medical Sciences. The population included all men and women working in emergency department with university degree of nursing, health expert, emergency medical technicians and nursing assistant who had at least one year of experience working in emergency department. The samples were selected based on simple random sampling method. First, a list of 40 emergency personnel was provided based on inclusion criteria. Then, each person was marked with a specific number from 1‐40. In the next procedure, the numbers were placed into a random number table. The researcher was present in three working shifts in the morning, afternoon and night for 4 weeks and communicated with the selected personnel. Those personnel who were not inclined to participate in the study were excluded.

### 3.2 Design

After explaining the objectives of the study and getting their consent letter, the participants were assured that this study does not affect their enhancement or position and is simply regarded as a research project. To comply with ethical considerations, the demographic questionnaire and checklists were anonymous and encoded. The sampling was based on observing the personnel during their work with respect to the mentioned treatment processes which summed up to 120 cases of intramuscular and intravenous injections, nasogastric intubation and bladder catheterization (30 cases for each) done by the researcher and project assistant in three working shifts for eight weeks until completing the number of the sample. Finally, the practical test was performed by an experienced nurse to avoid any bias in observations after data collection and observations.

### 3.3 Instrument

The data collection tools included a demographic questionnaire and observational checklists for each of the above‐mentioned processes. The demographic questionnaire was completed by interviewing the personnel. The questionnaire consists of the questions related to gender, age, experience, educational level, working shift and the type of treatment process. Observational checklists were provided according to the latest standards and searching the literature, Internet and clinical instructions. Each checklist had separate and different scores and was reported separately. Content reliability was measured to determine the reliability of the checklists, and simultaneous observation method was applied for determining the validity. In this regard, personnel's behaviour was observed by two observers simultaneously. Reliability of the tools was evaluated by Cronbach's alpha (α = 0.75). According to the study of Crutzen and Kuntsche (2013), the values greater or equal to 0.7 were considered as acceptable. The checklists related to compliance with safety principles in intramuscular injection had 20 questions with 20 scores. The obtained scores were classified into three levels of desirable (13.4–20), relatively desirable (6.8–13) and undesirable (0–6.7). Intravenous injection process has a 22‐question checklist with the total score of 22. The obtained scores were classified into three levels of desirable (14.7–22), relatively desirable (7.4–14.6) and undesirable (0–7.3). In addition, bladder catheterization had a 29‐question checklist with the total score of 29 and the obtained scores were classified into three levels of desirable (19.3–29), relatively desirable (9.7–19.2) and undesirable (0–9.6). Nasogastric intubation had a 19‐question worksheet with the total score of 19. Finally, the obtained scores were classified into desirable (12.7–19), relatively desirable (6.4–12.6) and undesirable (0–6.3) levels. In each worksheet, the “yes” answer had 1 score and “no” answer was considered as a negative score.

### 3.4 Data analysis

The obtained data were analysed by SPSS version 16.0 (IBM, USA). Descriptive analyses such as means, standard deviations and percentages were used to summarize the participants’ variables, along with their characteristics. Chi‐squared, independent *t* test and paired sample *t* test were used for the data analysis. The level of significance was considered as α < 0.05.

## 4 RESULT

The present study aimed to determine the level of applying safety standards in implementing therapeutic and caring procedures done in emergency department of Ganjavian Hospital of Dezful in 2015. Most of the staff were female, aged 20–29 years and had a bachelor's degree in nursing, with the work experience of 1–10 years (Table 1).

**Table 1:** Demographic information of participants

Procedures Variable	Muscular injections	Intravenous injections	Catheterization of the bladder	Nasogastric intubation
	Number (%)	Number (%)	Number (%)	Number (%)
Gender				
Female	30 (100.0)	19 (63.3)	19 (63.3)	17 (56.7)
Male	0 (0.0)	11 (36.7)	11 (36.7)	13 (43.3)
Age of personnel				
20–29	23 (76.7)	19 (63.3)	19 (63.3)	24 (80.0)
30–39	7 (23.3)	11 (36.7)	11 (36.7)	6 (20.0)
Education				
Nurse	29 (96.7)	27 (90.0)	28 (93.3)	22 (73.3)
Nurse aid	0 (0.0)	0 (0.0)	1 (3.3)	0 (0.0)
Emergency medical technicians	0 (0.0)	1 (3.3)	1 (3.3)	8 (26.7)
Health expert	1 (3.3)	2 (6.7)	0 (0.0)	0 (0.0)
Shift work				
Morning	7 (23.3)	11 (36.7)	11 (36.7)	13 (43.3)
Evening	11 (36.7)	8 (26.7)	1 (3.3)	5 (16.7)
Night	12 (40.0)	11 (36.7)	18 (60.0)	12 (40.0)
Work experience				
1–10	30 (100.0)	27 (90.0)	24 (80.0)	27 (90.0)
11–20	0 (0.0)	3 (10.0)	5 (16.7)	3 (10.0)
21–30	0 (0.0)	0 (0.0)	1 (3.3)	0 (0.0)

As shown in Table 2, the results of the safety standard compliance in the muscular injections procedure indicated that 63.3%, 33.3%., 3.3% of muscular injections were desirable, relatively desirable and undesirable, respectively. In addition, the results of the safety standard compliance in the intravenous injections procedure indicated that 86.7% of the intravenous injections were desirable while 13.3% were relatively desirable (Table 3).

**Table 2:** Frequency distribution of safety standards in the muscular injection procedure

Safety compliance in the muscular injections	Number	Percentage
Desirable	19	63.3
Relatively desirable	10	33.3
Undesirable	1	3.3
Total	30	100.0

**Table 3:** Frequency distribution of safety standards in the intravenous injection procedure

Safety compliance in the intravenous injections	Number	Percentage
Desirable	26	86.7
Relatively desirable	4	13.3
Undesirable	0	0.0
Total	30	100.0

Further, as indicated in Table 4, the results of the safety standard compliance in the catheterization of the bladder procedure showed that 90.0% of catheterization was desirable while 10.0% were relatively desirable. Finally, the results of the safety standard compliance in the nasogastric intubation procedure indicated that 80.0% of the nasogastric intubation was desirable while 20.0% was relatively desirable (Table 5). In general, based on the results, no statistically significant difference was observed between safety standard compliance during nasogastric intubation, bladder catheterization and intramuscular and intravenous injections procedures and some variables such as age, gender, experience, working shift and educational level.

**Table 4:** Frequency distribution of safety standards in the catheterization of the bladder procedure

Safety compliance in the Catheterization of the bladder	Number	Percentage
Desirable	27	90.0
Relatively desirable	3	10.0
Undesirable	0	0.0
Total	30	100.0

**Table 5:** Frequency distribution of safety standards in the nasogastric intubation procedure

Safety compliance in the nasogastric intubation	Number	Percentage
Desirable	24	80.0
Relatively desirable	6	20.0
Undesirable	0	0.0
Total	30	100.0

## 5 DISCUSSION

Based on the results, safety principles are satisfactorily met in intramuscular injection by 63.3% of personnel and 86.7% in intravenous injection, which is consistent with the results of Mahmoudi Markid and Feizi (2016) where the level of injection safety standards during and after injection complied by the nurses was desirable in most injections. However, in the study of Jalalinia et al. (2006), almost half of the injection was relatively desirable or undesirable. Nsubuga and Jaakkola (2005) indicated that the damage due to needle stick among the nurses and midwives was high (57%), which is mainly related to lack of training. Regarding nasogastric intubation procedure, safety principles followed by 20% of the personnel were relatively desirable while it was desirable among 80% of the personnel. In addition, Agha and Siddiqui (2011) reported that the incidence of mal‐placement of nasogastric tubes into the airways ranges from 0.3% to 15%, which is more common after chest trauma or mechanical ventilation, due to the need for adequate coordination of swallowing. Further, Sorokin and Gottlieb (2006) reported >2,000 feeding tube insertions over a 4‐year period throughout a major teaching hospital, nasogastric feeding tubes were mal‐positioned in 1.3%–2.4% of all insertions, and 28% of these malpositions resulted in pneumonia or pneumothorax. Thus, hospitals should adopt formal policies, procedures and monitoring to minimize catastrophic outcomes from a procedure erroneously assessed to be innocuous. Furthermore, the results revealed that safety principles were relatively desirable for 10% of people and desirable by 90% of people with respect to bladder catheterization. The results were not in line with the study of Adib‐Hajbaghery and Aghajani (2008), who indicated the care in bladder catheterization is in weak level from patients’ point of view. Bhatia, Daga, Garg, and Prakash (2010) showed that patients who underwent urinary catheters in emergency departments, especially female ones are exposed to high risk of urinary infection. In another study, Mosavian and Mashali (2004) reported that 43.6% of the patients had no symptoms of disease before urinary catheterization, but got bacteriuria after catheterization. In addition, some factors such as nonsterilized tools, catheterization by doctors, nurses or residents with different levels of training or catheterization in different places with different levels of contamination such as surgery rooms (Conway, Liu, Harris, & Larson, 2017) could result in increasing bacterial among the patients.

Further, Rostami and Tehrani (2011) concluded that the relative abundance of high‐risk behaviours due to no compliance with standards is unsatisfactorily high in the Hospital of Isfahan University of Medical Sciences. Laschinger and Leiter (2006) suggested that nurse administrators should develop strategies to create work environments which allow nurses to practice according to professional standards, which results in increasing work satisfaction, preventing burnout and assuring that patients are provided with safe effective high‐quality care. The results of the present study indicated that the level of compliance with safety principles had no statistically significant difference with age, work experience, level of education and working shift (*p* > 0.05) in any of four treatment procedures including nasogastric intubation, bladder catheterization and intramuscular and intravenous injections. Further, the highest rate of compliance was related to female personnel with 22–26 age ranges and 1–6 years of working experience. Regarding working shift, nasogastric intubation, bladder catheterization and intramuscular injections compliance were high in night shift, while it was high in morning shift for intravenous injection compliance. The study results of Ghasemi et al. (2009) on wounds due to needle stick indicated no statistically significant relationship between age and safety principles compliance (*p* = 0.71). Furthermore, Azarbarin (2008) indicated no statistically significant relationship between age and working experience with the level of compliance with safety principles in intramuscular injection. The study results of Jalalinia et al. (2006) reported a statistically significant difference between gender and methods used for safe injection (*p* = 0.002), which may be related to the accuracy of women. In another study, Rostami and Tehrani (2011) observed a statistically significant relationship between age, gender, job and working experience with compliance with standards, which is not in line with the results of present results. In these studies, the role of age, gender, educational level, working experience, working shift and educational degree in the level of compliance with safety principles during treatment procedures among emergency personnel with weaker levels of following safety principles and standards emphasized the need for designing and programming further training and education, especially among the group. Finally, it is recommended to use more samples for obtaining more accurate results.

### 5.1 Limitation of the study

The present study had some limitations such as low sample size although the samples had the equal chance to enter the study. However, conducting research with more population based on more procedures may provide different results. In addition, some factors include large number of referring patient, especially in night shifts. Thus, less attention to nurses for implementing safety standards in caring and therapeutic procedures, nurses’ fatigue, lack of competent nurses and the need to take immediate action in critical patient can influence the study results. In spite of these limitations, the questionnaires were completed accurately and there was no problem with the implementation method.

## 6 CONCLUSION

Based on the results, the most observed procedures were in a desirable range. However, according to the World Health Organization, full compliance (100%) with these standards is necessary since the slight failure in compliance results in transmitting infection and illness. Thus, the standards and proper principles of conducting procedures should be desirably complied and attempts should be made to increase the degree of applying these safety standards to prevent infectious transmission damage to ourselves and the patient, and maintaining the patient's safety, all personnel involved in the care and treatment, especially nurses. In addition, hand hygiene was poor in all four procedures. Health education is regarded as the nurse's major role in infection prevention. Clients and caregivers need to learn about effective hand washing, use of gloves, handling lines and disposal of wastes and soiled dressing (Berman, Snyder, & Frandse, 2016). Regarding the importance of hand hygiene in reducing microbial transmission and its effect in reducing hospital infections and mortality rate, further studies should be conducted with respect to the assessment of the cause of noncompliance with hand hygiene based on existing models (e.g., Health Belief Model) among the personnel such as doctor, nurse, nursing assistant, practical nurse and the like.

## ETHICAL APPROVAL

This study was approved by the Ethics Committee affiliated to Dezful University of Medical Sciences (Ethics Code: IR.DUMS.REC 1395.17). In this study, researchers were committed to ethical issues of obtaining informed consent from the participants, respect for voluntary participation and inform the participants about the purpose of the study.

## CONFLICT OF INTEREST

I have no conflicts of interest to declare.
